# The Art of Prescribing

**Published:** 1867-08

**Authors:** 


### The Art of Prescribing.

It is to be doubted whether the improvement in the art of prescribing has kept pace with that of our knowledge of drugs and chemical combinations. Be that as it may, in some of the prescriptions that are handed to the pharmaceutist to dispense, the most incongruous intermixture of remedies, most dissimilar and contradictory in action, is ordered; whilst chemical considerations are set at defiance, and no regard is paid to the often complex changes that must take place upon the admixture of several different preparations, whose original properties are often completely altered. The real intentions of the prescriber are thus defeated, and the cure indirectly delayed.

Mr. Daniel Hanbury recently discussed this subject at a meeting of the Pharmaceutical Society. He referred first of all to *unchemical formulæ*, giving as illustrations the combination of chloride of barium, sulphate of iron, and extracts of gentian; the chloride being thus rendered inert; and, secondly, a prescription containing iodide of potassium, bicarbonate of potash, citrate of iron and quinine, ammoniated tincture of valerian, and water, the result being the production of a frothy white precipitate of quinia, forming a mass suitable for pills. And this exemplifies a point not sufficiently well known—that quinia does not combine with ammonia or an alkaline carbonate. *Unexpected combinations* some times result, of which the following is an example:—A prescription was written for a mixture, of which the more essential ingredients were Rochelle salts and calcined magnesia; this was taken without particular remark until a dose was swallowed from a bottle of the medicine which had been prepared some weeks. The effect was so disagreeable, and the taste so caustic, that the patient believed some error had been committed, and special inquiries resulted in the explanation that the calcined magnesia, by prolonged contact with alkaline tartrates, had gradually abstracted their tartaric acid, leaving the alkalies in a free and caustic coadition. *Ill-contrived formulæ* are very frequent; but these are not of serious consequence. *Undue concentration of medicines*, Mr. Hanbury showed, was fraught with special inconvenience to the pharmaceutist, and with risk to the patient. The economical considerations did not outweigh those of safety and efficacy in the action of drugs; and he quoted actual prescriptions in which the liquid was incapable of holding in solution the alkaline salts ordered, so that they crystalized out or remained as a dense white mass, which could not in some cases be shaken up. One contained chlorodyne, bi-borate of soda, spirits of camphor, aromatic ammonia, and sulphuric ether; in this the addition of the borax to the other ingredients occasioned the separation of a sticky mass, which adhered to the inside of the bottle, and prevented the administration of the proper dose. One prescription noticed contained six grains of corrosive sublimate, a second an ounce of arsenical solution with three drachms of tincture of aconite—manifestly liable to mischief from the common carelessness of patients in measuring doses. These are not, however, to be compared to other more dangerous forms of prescriptions; to those, for instance, in which a bottle containing about 150 doses of the strongest tincture of aconite is ordered, with directions to take a dose every three hours; or where nearly 100 doses of strychnine are placed in the patient's hands at once; or a five-weeks' supply of the same alkaloid in a ten-drachm mixture, with complicated directions; or two grains of arsenious acid are ordered to be dissolved in two ounces of syrup of ginger—a vehicle which, being extremely palatable, would convey the idea that the drops were more or less harmless. Such instances as these, by no means uncommon, are excessively objectionable; as Mr. Hanbury observed, they are reprehensible for the sake of the patient, who is furnished with a large supply of potent, or it may be dangerous, medicine, which is to be taken for a long period, almost according to his own pleasure and judgment, and especially for the sake of the pharmaceutist, not so much on account of the diminished remuneration, as of the very serious risk of error and accident, which may at any time place him in an unpleasant position.—*London Lancet.*

